# Rapid and Sensitive Recombinase Polymerase Amplification Combined With Lateral Flow Strip for Detecting African Swine Fever Virus

**DOI:** 10.3389/fmicb.2019.01004

**Published:** 2019-05-15

**Authors:** Faming Miao, Jingyuan Zhang, Nan Li, Teng Chen, Lidong Wang, Fei Zhang, Lijuan Mi, Jinxia Zhang, Shuchao Wang, Ying Wang, Xintao Zhou, Yanyan Zhang, Min Li, Shoufeng Zhang, Rongliang Hu

**Affiliations:** ^1^Institute of Military Veterinary Medicine, Academy of Military Medical Science, Changchun, China; ^2^College of Life Science, Ningxia University, Yinchuan, China

**Keywords:** African swine virus, on site detection, recombinase polymerase amplification, lateral flow strip, RPA-LFD

## Abstract

African swine fever virus (ASFV), the etiological agent of African swine fever (ASF), a hemorrhagic fever of domestic pigs, has devastating consequences for the pig farming industry. More than 1,000,000 pigs have been slaughtered since 3 August 2018 in China. However, vaccines or drugs for ASF have yet to be developed. As such, a rapid test that can accurately detect ASFV on-site is important to the timely implementation of control measures. In this study, we developed a rapid test that combines recombinase polymerase amplification (RPA) of the ASFV p72 gene with lateral flow detection (LFD). Results showed that the sensitivity of recombinase polymerase amplification with lateral flow dipstick (RPA-LFD) for ASFV was 150 copies per reaction within 10 min at 38°C. The assay was highly specific to ASFV and had no cross-reactions with other porcine viruses, including classical swine fever virus (CSFV). A total of 145 field samples were examined using our method, and the agreement of the positive rate between RPA-LFD (10/145) and real-time PCR (10/145) was 100%. Overall, RPA-LFD provides a novel alternative for the simple, sensitive, and specific identification of ASFV and showed potential for on-site ASFV detection.

## Introduction

African swine fever (ASF) is a severe viral disease that manifests clinical symptoms of hemorrhagic fever caused by African swine fever virus (ASFV) and can result in case fatality rates of up to 100% in domestic pigs, depending on the virus strain (Galindo and Alonso, 2017). ASF, which was first identified by Montgomery (1921) in Kenya in the 1920s, made its first incursion into Europe via two successive entries into Portugal (in 1957 and again in 1960), spreading rapidly throughout Western Europe and then to South America and the Caribbean (Michaud et al., 2013). It was eventually eradicated by the mid-1990s, with the exception of Sardinia (Mur et al., 2016). However, since the second major incursion of the disease, initially into Georgia in 2007, ASF has spread to Eastern Europe and Russia (Rowlands et al., 2008; Revilla et al., 2018). The virus has continued to spread worldwide, including China. Since the first ASF case emerged in China in August 2018, more than 100 cases have been recorded in 25 provinces (Zhou et al., 2018).

African swine fever normally presents with non-specific symptoms, including fever, anorexia, vomiting, and diffused hemorrhage in superficial skin. Post-mortem examination shows pericardial effusion, kidney enlargement, lymphadenectasis, and darkened and enlarged spleen. All these features are indistinguishable from those seen in classical swine fever virus (CSFV) infection (Tauscher et al., 2015; Li and Tian, 2018). At present, no effective treatment or vaccine for ASFV is available, and disease control is based mainly on animal slaughtering and strict sanitary measures (Cisek et al., 2016; Rock, 2017). Rapid laboratory diagnosis is important for timely triage and confirmation to control and preventing this disease because of its rapid progression to death and spread.

Molecular tools based on the detection of the genetic information of ASFV have become more widely accepted for ASF diagnosis (Oura et al., 2013). Polymerase chain reaction (PCR) and real-time PCR techniques have provided a supportive method to post-mortem ASF diagnosis (Aguero et al., 2003; Zsak et al., 2005; Fernandez-Pinero et al., 2013); however, they cannot be used for field (on-site) detection in pig farms. Recently, Liu et al. (2019) reported that the improved real-time PCR assay using a Universal Probe Library (UPL) probe could be applied to ASFV molecular diagnosis under field conditions. Due to limitations of the battery-powered real-time PCR instrument, it can process only a moderate number of samples. Isothermal recombinase polymerase amplification (RPA) has been successfully used to detect multiple viral pathogens, including infectious bovine rhinotracheitis virus (Hou et al., 2017), bovine coronavirus (Amer et al., 2013), Ebola virus (Yang et al., 2016), bovine viral diarrhea virus, or foot-and-mouth disease virus (Wang et al., 2018). As reports of ASFV detection by RPA are limited, we aimed to develop and evaluate a rapid detection tool that combines immunochromatographic strip tests, more commonly referred to as lateral flow devices (LFDs), with RPA targeting the conserved ASFV p72 gene.

## Materials and Methods

### Ethics Statement

This study was conducted as part of the surveillance of the ASF outbreak in China. Samples were collected for ASF testing and surveillance under the agreement between the Ministry of Agriculture and Rural Affairs of the Chinese Government and farm owners. Sample collecting treatment was conducted in accordance with the protocols for viral hemorrhagic fever under the urgent interim guidance for case management established by the World Organization for Animal Health. The protocol for this study was approved by the ethics committee of the Military Veterinary Research, Academy of Military Medical Sciences. Samples were collected by animal centers for disease control and prevention of Jilin Province. The viruses were inactivated in the BSL-3 Lab, and the inactivated samples were transferred to the BSL-2 Lab for genomic DNA extraction and detection.

### Nucleic Acid Extraction

For standard plasmids, a plasmid extraction kit was used in accordance with the manufacturer’s instructions (Axygen, United States). For field samples, tissue homogenates were prepared in formaldehyde, and the inactivated virus homogenate was subjected to DNA extraction by using a nanomagnetic bead adsorption kit (Bailing Biotechnology, Beijing Co., Ltd., China). For the whole extraction procedure, lysis, adsorption, and washing were performed, and elution with 50 μL of RNase-free water was conducted to dissolve the DNA. The extracted DNA was then stored at -20°C.

### Design of the Primers and Probes of RPA

**FIGURE 1:**
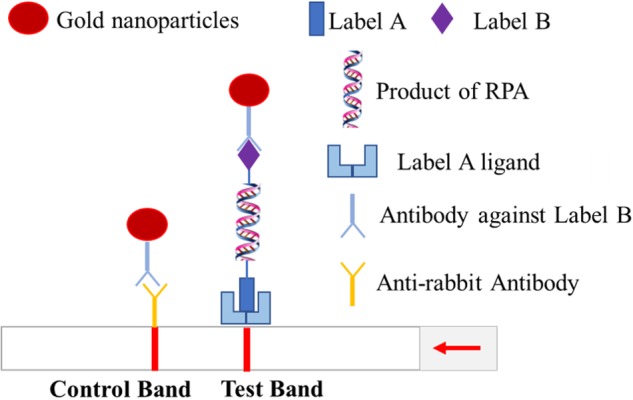
Schematic of RPA-LFD assay.

The p72 gene was first amplified in the RPA reaction system comprising the designed primers (labeled biotin) and a 5-Carboxyfluorescein (FITC) labeled-probe, and the expected amplicons were labeled with 5′-FITC and 3′-biotin at the ends. Then, the amplified products were recognized by anti-biotin and anti-FITC monoclonal antibodies on the test line, where gold nanoparticles were prefixed (Figure 1).

The whole genome sequence of a p72 genotype II virus—ASFV-SY18, which was sequenced in our lab (GenBank accession number MH766894)—was used as the reference sequence for RPA primer selection. RPA sense primer (5′-AAGAAGAAAGTTAATAGCAGATGCCGATACCAC-3′), RPA manti-sense primer (5′-Biotin-GCTCTTACATACCCTTCCACTACGGAGGCAATG-3′), and RPA probe primer (5′-FAM-TGGGTTGGTATTCCTCCCGTGGCTTCAAAGCAAAG[THF]TAATCATCATCGCAC-P-3′) were designed by using TwistDx NFO RPA kits (Cambridge, United Kingdom) in accordance with the manufacturer’s guidelines.

### Real-Time qPCR Assay

TaqMan PCR was performed to detect ASFV p72 in an Mx3000P multiplex quantitative PCR system (Stratagene) in accordance with the manufacturer’s instructions. Real-time PCR primers for p72 were used as described before (King et al., 2003). The sequences spanning the RPA amplification region were obtained through PCR and cloned into the pMD19-T plasmid to generate pMD19-p72. TaqMan runs of the experimental samples involved at least three replicates with no-template or no-primer control and the combination of the primers. The PCR conditions were 95°C for 2 min and 40 cycles of 95°C for 15 s and 60°C for 60 s. A standard curve was generated from serially diluted p72 recombinant plasmids of known copy numbers.

### Development of the ASFV RPA-LFD Assay

A pair of primers and RPA probe primer was designed to specifically detect ASFV circulating strains in China. The RPA assay was conducted in a 50 μL system by using a TwistAmp NFO kit (TwistDx^TM^, Cambridge, United Kingdom). All the reagents (500 nM RPA primers, 120 nM RPA probe, 4 × rehydration buffer, and purified H_2_O) except the template or the sample DNA and magnesium acetate were prepared in a master mix, which was aliquoted into each tube of a 0.2 mL tube strip containing a dried enzyme pellet. Magnesium acetate (2.5 μL) was pipetted slowly into the tube lids, and subsequently, 2 μL of the sample DNA was added to the tubes, the lids were closed, and magnesium acetate was centrifuged into the tubes by using a mini-spin centrifuge. The tubes were immediately used for isothermal amplification. ASFV-RPA detection was combined with an LFD (Yoshida, Co., Ltd., China). Then, 2 μL of the amplified product was added to the sample pad, and the dipstick was inserted into 100 μL of the sample buffer for 3–5 min.

### Sensitivity and Specificity of ASFV RPA-LFD Assays

A dilution range of 10^0^ to 10^5^ copies per reaction of pMD19-p72 recombinant plasmid was used to evaluate the sensitivity of recombinase polymerase amplification with lateral flow dipstick (RPA-LFD), and the amplicons were evaluated through agarose gel electrophoresis. The reactions were evaluated with PRRSV/CH1R, CSFV/Shimen, PRV/JL14, JEV/SA14-14-2, PEDV/CV777, and PCV2b-SD12 to examine the specificity of the developed RPA-LFD assay.

## Results

### Sensitivity and Specificity of TaqMan ASFV Real-Time Assay

**FIGURE 2:**
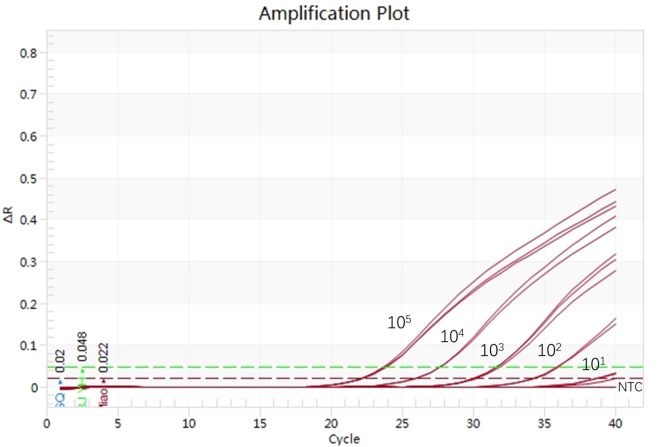
Evaluation of the sensitivity of ASFV TaqMan real-time PCR.

The thermo-reaction procedures were optimized after different primers, probe combinations, and thermo-cycles were run in the real-time PCR system to evaluate the sensitivity of TaqMan ASFV real-time PCR assay. The real-time PCR conditions that yielded the highest amplification efficiency were 94°C for 5 min and 40 cycles of 94°C for 30 s and 55°C for 30 s by using the primer combination reported by King et al. (2003). The real-time PCR assay was sufficiently sensitive to detect 10^2^ copies per reaction (Figure 2). No cross-reactions with PRRSV/CH1R, CSFV/Shimen, PRV/JL14, JEV/SA14-14-2, PEDV/CV777, and PCV2b-SD12 were observed, and a positive signal was detected in ASFV-SY18 and ASFV-JL18.

### Optimization of the Reaction Condition of RPA-LFD for ASFV Detection

**FIGURE 3:**
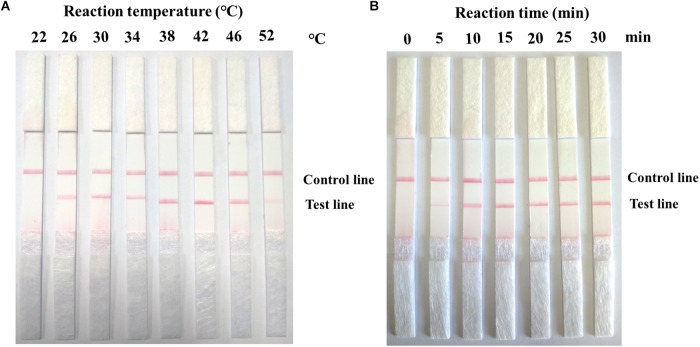
Optimal detection conditions of ASFV RPA-LFD assay. **(A)** The assay works from 26°C to 46°C. **(B)** After 5 min of amplification, the test line is visible on the lateral flow dipstick.

In this study, 10^5^ copies of the ASFV-positive pMD19-p72 plasmid were used as a template to determine the optimal RPA reaction temperature. The reaction mixture was incubated at eight different temperatures (22°C, 26°C, 30°C, 34°C, 38°C, 42°C, 46°C, and 52°C) for 15 min. The detection result showed that the test line was short at 26°C and 52°C, and the test line density did not enhance significantly from 30°C to 46°C (Figure 3A). In this study, 10^5^ copies of the ASFV-positive plasmid were used as a template to determine the optimal RPA reaction time. In Figure 3B, the RPA assay tested positive for ASFV in 5 min. The measured DNA band density revealed that the DNA yield doubled after a reaction time of 10 min and increased slightly after 15 min. The intensity of the DNA bands in agarose gel did not significantly change at reaction times of 15, 20, 25, and 30 min. This finding indicated that the RPA reactions during the remaining parts of this study were completed after 12 min. However, the false positive test lines appeared when the reaction time was more than 15 min (invalid result). Therefore, 10 min was considered the optimum reaction time for the RPA-LFD assay, and the assay worked from 26°C to 46°C. In terms of convenience and safety, 38°C was chosen as the optimum reaction temperature.

### Sensitivity and Specificity of ASFV RPA-LFD Assay

**FIGURE 4:**
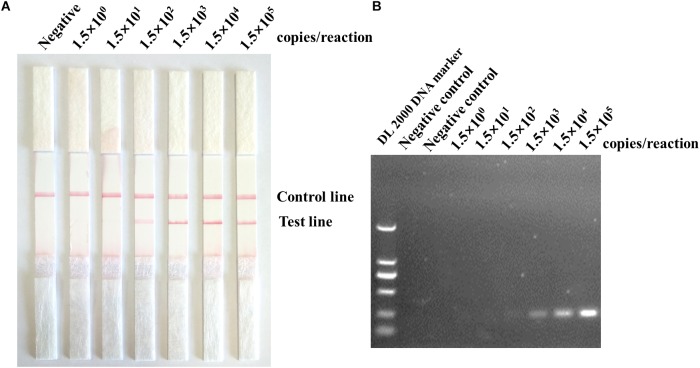
Evaluation of the sensitivity of ASFV RPA-LFD assay. **(A)** In the lateral flow format (ASFV RPA-LFD), the sensitivity was 150 copies per reaction of the standard plasmid. **(B)** Positive ASFV RPA reaction products could be detected on the stained agarose gel (2%).

**FIGURE 5:**
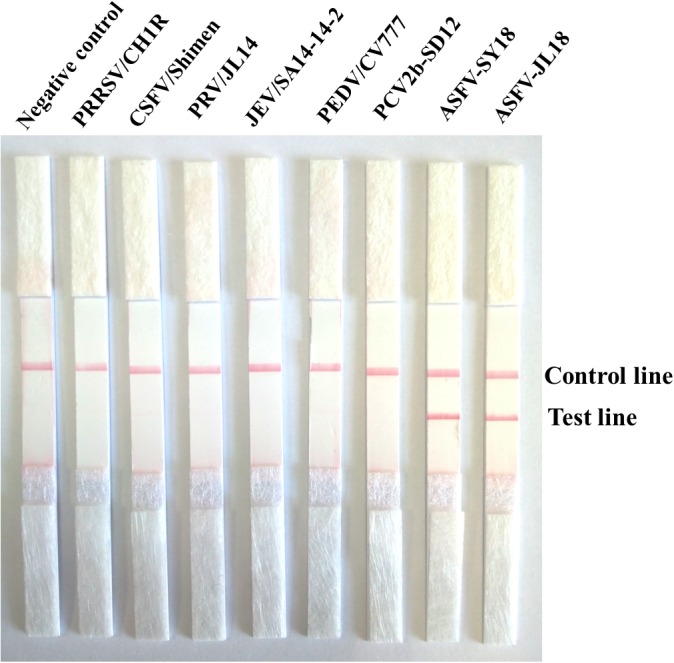
Specificity test results of ASFV RPA-LFD assay involving Nano-magnetic bead DNA extraction from ASFV and other porcine viruses.

The sensitivity results showed that the detection limit of the ASFV RPA-LFD assay was 10^2^ copies per reaction of the recombinant plasmid pMD19-p72. The amplicon in the ASFV RPA-LFD assay (230 bp) was also tested through subsequent visualization with 2% agarose gel-electrophoresis after purification, and the sensitivity of RPA based on gel-electrophoresis visualization was 10^3^ copies per reaction (Figure 4). In terms of the specificity of ASFV RPA-LFD assay, no cross-reactions with other important viruses of pigs were observed (Figure 5), and this observation was confirmed by agarose gel electrophoresis.

### Performance of ASFV RPA-LFD Assay on Clinical Samples

The 145 samples included 90 spleen samples and 55 blood samples of animals (Jilin CDC). Of these samples, 5 spleen tissues and 5 blood samples were positive for ASFV nucleic acid as detected by RPA-LFD. TaqMan real-time PCR showed that 10 samples were positive. The RPA-LFD detection results were consistent with those of real-time PCR. RPA-LFD revealed that the samples showed a light test line and high Cq values. Of these samples, one tested sample, which showed with a short test line or no test line, was rotten, and the Cq value in real-time PCR was 38, indicating weakly positive result. Although the detection results between the two methods were consistent (Table 1), the RPA-LFD assay was more rapid than real-time PCR. In particular, the whole RPA-LFD assay yielded results in 30 min, whereas real-time PCR generated results approximately 2 h later.

**Table 1:** Parallel test results between RPA-LFD and TaqMan real-time PCR for clinical samples.

Sample types	Sample numbers	Results (Positive/Negative)	
		Real-time PCR	RPA-LFD
Blood tissues	55	5/50	5/51
Spleen tissues	90	5/85	5/85


## Discussion

The average time from symptom onset to outcome in ASF is 6–20 days; however, the pig farms were usually in rural areas or even in a mountainous area far away from the city laboratory, which required more time for sampling and transportation. Once diagnosis and timely treatment of suspected ASF pigs is delayed, the risk of ASF exposure to other pig farms was increased. In the current outbreak, laboratory testing with TaqMan real-time PCR is being widely used in affected areas. However, requirements—including a sophisticated thermocycler, complex sample treatment procedures, and expensive reaction instruments—have limited its applications in point-of-care testing (King et al., 2003).

Different isothermal molecule amplification assays, including loop-mediated isothermal amplification assay (LAMP) (James et al., 2010), polymerase cross-linking spiral reaction (Wozniakowski et al., 2017), cross-priming amplification method (Fraczyk et al., 2016), chimeric DNA/LNA-based biosensor (Biagetti et al., 2018), and droplet digital PCR (Wu et al., 2018), have been developed as a rapid, simple, and cost-effective alternative to PCR-based molecule assay. RPA has several advantages. For example, initial heating for DNA denaturation is not required, and test conditions (38°C within 20 min) are easily implemented. LAMP assay for ASFV detection requires a long time (60 min) and high temperature (62°C-65°C) (James et al., 2010). By comparison, RPA assay takes less than 20 min at 38°C to complete. As such, RPA assay is simpler and more easily used than LAMP assay.

Wang et al. (2017) reported the field validation of real-time RPA for ASFV, although this technique can specifically detect ASFV plasmid with a detection limit of 10^2^ DNA copies per reaction. However, this detection assay is based on an expensive scanner device. Detection methods for actual clinical samples have yet to be designed, so the application of this test to clinical samples is limited. Gao et al. (2018) developed cross-priming amplification combined with immune-chromatographic strips for the rapid on-site detection of ASFV, but its reaction conditions include 60 min at 59°C and six primers for a reaction system, thereby causing non-specific amplification. The use of lateral flow assays combined with a monoclonal antibody against p72 protein of ASFV can be used to detect p72 viral and recombinant protein or inactivated culture virus (Sastre et al., 2016); however, the sensitivity of the assay is 100-fold lower than genomic amplification.

Recombinase polymerase amplification with lateral flow dipstick overcomes the technical difficulties posed by current amplification methods; for example, it operates at a low and constant temperature, does not require the thermal denaturation of templates, and does not rely on an expensive thermocycler (Aguero et al., 2003). In combination with a commercially magnetic nanobead-based DNA extraction kit, this approach can be applied to on-site testing or rapid diagnosis under poorly equipped conditions. RPA is highly resistant to crude samples, suggesting that it can be used for on-the-spot field testing with crude nucleic acid extraction. In an RPA-NFO reaction system, NFO cleaves the primer of the probe at the THF position and effectively deblocks the probe, thereby generating a new 3′ hydroxyl group that can act as a primer for polymerase extension; thus, the probe is transformed into an extension primer with an increased specificity of amplification (James and Macdonald, 2015).

The potential defect of RPA-LFD assay for ASFV is that it may carry over contaminants in fields because its tube must be opened after amplification is completed. Therefore, precautions should be taken. For example, reaction tubes should be carefully opened and closed, gloves should frequently be changed, the progress of pre- and post-RPA amplification should be separated, and reaction time should be shortened if possible. The replacement of dTTP with dUTP may help prevent such carryover contamination as demonstrated in other nucleic amplified system assays.

In summary, we evaluated a rapid RPA-LFD test for the rapid diagnosis of ASFV. The superior performance of RPA-LFD for ASFV and its consistent detection results with those of real-time PCR indicated its appropriateness for laboratory diagnosis and that it has great potential for on-site testing of ASFV circulating in China.

## Data Availability

The raw data supporting the conclusions of this manuscript will be made available by the authors, without undue reservation, to any qualified researcher.

## Ethics Statement

This study was conducted as part of the surveillance of the ASF outbreak in China. Samples were collected for ASF testing and surveillance under the agreement between the Ministry of Agriculture and Rural Affairs of the Chinese Government and farm owners. Sample collecting treatment was conducted in accordance with the protocols for viral hemorrhagic fever under the urgent interim guidance for case management established by the World Organization for Animal Health. The protocol for this study was approved by the ethics committee of the Military Veterinary Research Institute, Academy of Military Medical Sciences. Samples were collected by animals centers for disease control and prevention of Jilin Province. The viruses were inactivated in the BSL-3 Lab, and the inactivated samples were transferred to the BSL-2 Lab for genomic DNA extraction and detection.

## Author Contributions

ML, SZ, and RH designed the experiments. FM, JYZ, NL, TC, LW, FZ, LM, JXZ, SW, YW, XZ, and YZ performed the experiments. ML and SZ analyzed the data. FM, JYZ, and RH wrote the manuscript.

## Conflict of Interest Statement

The authors declare that the research was conducted in the absence of any commercial or financial relationships that could be construed as a potential conflict of interest.
